# Submacular Hemorrhage Management: Evolving Strategies from Pharmacologic Displacement to Surgical Intervention

**DOI:** 10.3390/jcm15020469

**Published:** 2026-01-07

**Authors:** Monika Sarna, Arleta Waszczykowska

**Affiliations:** 1 Department of Ophthalmology, Wojewódzkie Centrum Szpitalne Kotliny Jeleniogórskiej, 58-506 Jelenia Góra, Poland; monikasarna27@gmail.com; 2 Department of Ophthalmology, Medical University of Lodz, Kopcinskiego 22, 90-153 Lodz, Poland

**Keywords:** submacular hemorrhage, pneumatic displacement, pars plana vitrectomy, rtPA, anti-VEGF therapy, neovascular AMD, polypoidal choroidal vasculopathy, surgical management

## Abstract

**Background**: Submacular hemorrhage (SMH) is a vision-threatening condition most associated with neovascular age-related macular degeneration (nAMD), although it may also arise from polypoidal choroidal vasculopathy, pathological myopia, retinal vascular diseases, trauma, and systemic factors. Rapid management is essential because subretinal blood induces photoreceptor toxicity, clot organization, and fibroglial scarring, leading to irreversible visual loss. The choice and urgency of treatment depend on hemorrhage size, duration, and underlying pathology, and the patient’s surgical risk category, which can influence the invasiveness of the selected procedure. This review aims to provide an updated synthesis of recent advances in the surgical and pharmacological management of SMH, focusing on evidence from the past five years and comparing outcomes across major interventional approaches. **Methods**: A narrative review of 27 recent clinical and multicentre studies was conducted. The included literature evaluated pneumatic displacement (PD), pars plana vitrectomy (PPV), subretinal or intravitreal recombinant tissue plasminogen activator (rtPA), anti-VEGF therapy, and hybrid techniques. Studies were analyzed about indications, surgical methods, timing of intervention, anatomical and functional outcomes, and complication and patient risk stratification. **Results**: Outcomes varied depending on the size and duration of hemorrhage, as well as the activity of underlying macular neovascularization. PD with intravitreal rtPA was reported as effective for small and recent SMH. PPV combined with subretinal rtPA, filtered air, and anti-VEGF therapy demonstrated favorable displacement and visual outcomes in medium to large hemorrhages or those associated with active nAMD. Hybrid techniques further improved clot mobilization in selected cases. Across studies, delayed intervention beyond 14 days correlated with reduced visual recovery due to blood organization and photoreceptor loss. Potential risks, including recurrent bleeding and rtPA-associated toxicity, were reported but varied across studies. **Conclusions**: Management should be individualized, considering hemorrhage characteristics and surgical risk. Laser therapy, including PDT, may serve as an adjunct in the perioperative or postoperative period, particularly in PCV patients. Early, tailored intervention typically yields the best functional outcomes.

## 1. Introduction

Submacular hemorrhage (SMH) represents a vision-threatening complication that requires timely and individualized medical intervention depending on the patient’s clinical presentation and general condition. The predominant underlying etiology of SMH is neovascular age-related macular degeneration (nAMD) [1,2,3,4]. According to the current nomenclature of neovascular AMD and polypoidal choroidal vasculopathy (PCV), the latter may be classified as a subtype of type I macular neovascularization (MNV) when associated with AMD, although PCV can also arise independently within the pachychoroidal disease spectrum. Other less frequent causes of SMH include pathological myopia-related complications, ocular trauma [1], diabetic retinopathy, retinal vein occlusion, coagulation abnormalities, systemic hypertension, the Valsalva maneuver [2], and rupture of a retinal macroaneurysm [2,3].

The choice and invasiveness of treatment should consider not only hemorrhage size and duration but also the patient’s surgical risk category. High-risk patients may be better suited for minimally invasive PD, while low-risk patients can tolerate more extensive PPV-based approaches. Prompt diagnosis and rapid initiation of treatment are crucial, due to the potential toxicity of subretinal blood to retinal structures, which may result in irreversible visual acuity deterioration and, in severe cases, permanent blindness. Various treatment modalities for SMH have been described in the literature, including both conservative approaches (e.g., intravitreal administration of fibrinolytic agents and expansile gases to displace the clot) and numerous surgical techniques [e.g., pars plana vitrectomy (PPV) combined with recombinant tissue plasminogen activator (rtPA), mechanical evacuation of the hemorrhage, or retinal pigment epithelium (RPE) transplantation].

Despite extensive research, several controversies and diverging hypotheses remain unresolved. There is no consensus regarding the optimal therapeutic strategy, particularly when comparing minimally invasive pneumatic displacement with more complex PPV-based approaches. The potential retinal toxicity of rtPA continues to be debated, with studies reporting conflicting outcomes. Moreover, the ideal timing of intervention remains uncertain: although early surgery is often advocated, excessively early procedures may increase the risk of rebleeding, whereas delayed management promotes clot organization and limits visual recovery.

Experimental and clinical studies indicate that the “acceptable time window” for surgical or pharmacological intervention is typically within 7 to 14 days from the onset of bleeding, beyond which the risk of irreversible photoreceptor loss and subretinal fibrosis significantly increases [2,3,4].

This review aims to provide an updated and integrative synthesis of recent advances in the surgical and pharmacological management of SMH, with a focus on evidence published over the past five years. By systematically comparing outcomes and complication rates across major interventional approaches, it highlights evolving surgical preferences, timing-dependent prognosis, and procedural nuances that remain underrepresented in prior literature. Overall, current evidence suggests that therapeutic decisions should be individualized, and that outcomes depend largely on hemorrhage size, timing of intervention, and underlying neovascular activity.

## 2. Materials and Methods

This narrative review was conducted to summarize recent advances in the surgical and pharmacological management of submacular hemorrhage (SMH), with particular emphasis on pars plana vitrectomy (PPV), pneumatic displacement (PD), and hybrid approaches combining subretinal recombinant tissue plasminogen activator (rtPA), anti-VEGF therapy, and filtered air. The review focused on evidence published within the last five years to reflect current clinical practice and emerging trends.

A literature search was performed in PubMed, Web of Science, and Scopus between January 2019 and December 2024. Patient risk stratification was extracted when reported, to evaluate its influence on surgical choice and outcomes. Data included treatment indications, techniques, pharmacologic adjuncts, anatomical and functional outcomes, complications, and use of laser therapy, including PDT. The following keywords and their combinations were used: submacular hemorrhage, subretinal hemorrhage, pars plana vitrectomy, pneumatic displacement, rtPA, anti-VEGF, polypoidal choroidal vasculopathy, and macular neovascularization. Only articles published in English and involving human subjects were included. Additional references were identified by screening the bibliographies of relevant publications.

As this is a narrative review, no formal systematic review protocol, PRISMA flowchart, meta-analysis, or quantitative comparison was performed. Studies were selected based on their relevance, methodological clarity, sample size, clinical applicability, and contribution to understanding current SMH treatment strategies. Preference was given to multicenter studies, real-world evidence, and research directly comparing interventional approaches.

Extracted information included treatment indications, surgical techniques, pharmacologic adjuncts, anatomical and functional outcomes, and reported complications. Findings were synthesized qualitatively to highlight areas of consensus, controversy, and evolving clinical practice patterns.

Artificial intelligence (ChatGPT, OpenAI; GPT-5.2 version) was used solely to assist in language refinement and editing after the authors generated the full scientific content. All factual data, interpretation, and conclusions were derived independently by the authors.

## 3. Results

### 3.1. Surgical Management

The surgical approach to SMH has progressively evolved in parallel with advances in microsurgical techniques and the development of modern pharmacological therapies. The current primary goal is the maximal preservation of the macula and surrounding retinal structures, while aiming to minimize invasiveness. Although no universally accepted criteria clearly define indications for surgical intervention in the existing literature, the decision to proceed with surgery should be individually tailored to hemorrhage characteristics and patient surgical risk.

High-risk patients often receive PD ± intravitreal rtPA to minimize surgical stress.

Medium-risk patients may undergo PPV with subretinal rtPA or triple therapy (rtPA + anti-VEGF + filtered air).

Low-risk patients can tolerate more invasive PPV procedures including retinotomy or retinectomy for large or chronic SMH.

From a pathophysiological standpoint, submacular hemorrhage initiates several potentially harmful processes; however, their severity and clinical consequences vary widely depending on the hemorrhage’s size, thickness, duration, and underlying etiology. The accumulation of blood beneath the macula can mechanically separate the neurosensory retina from the RPE, and hemoglobin and iron degradation products may contribute to oxidative stress, inflammation, and cytotoxicity. These mechanisms have been implicated in photoreceptor dysfunction and, in some cases, in the development of subretinal fibrosis.

Importantly, these processes do not invariably lead to irreversible central vision loss, and smaller or thinner hemorrhages—particularly when associated with neovascular AMD treated promptly with anti-VEGF therapy—may show meaningful visual recovery without the need for surgical or displacement procedures. Reported variability in outcomes highlights the influence of multiple factors, including hemorrhage configuration, chronicity, and the activity of the underlying choroidal neovascularization.

rtPA binds to fibrin to form a complex that converts plasminogen into plasmin, facilitating enzymatic clot lysis. For selected cases, earlier displacement may reduce the duration of contact between blood and photoreceptors, potentially limiting secondary damage, although this benefit must be balanced against procedural risks.

It is important to note that most of the available studies were retrospective or prospective non-randomized analyses. Surgical technique selection (PD vs. PPV) was often based on surgeon preference and the size, thickness, and location of the hemorrhage rather than on standardized random assignment. Consequently, potential bias related to case selection and surgeon experience should be considered when interpreting the outcomes of these studies [2,3,4,5,6,7,8].

Some reports suggest that a hemorrhage exceeding one disk diameter (DD) in size [3] and greater than 100 µm in thickness, as measured by optical coherence tomography (OCT) [4], may be as criteria for surgical intervention.

Indications for surgery include large and/or thick hemorrhages that are unlikely to undergo spontaneous resorption within an acceptable timeframe, hemorrhages associated with active choroidal neovascularization, and cases with coexisting structural damage to the macula, such as intraretinal fibrosis, epiretinal membranes (ERM), or rhegmatogenous retinal detachment (RD).

Surgical techniques used in SMH management exhibit substantial heterogeneity, reflecting both the absence of standardized treatment protocols and the complex etiology of the condition. The literature outlines several principal surgical strategies, ranging from minimally invasive methods aimed at clot displacement using intraocular gas and fibrinolytics, to more advanced vitrectomy-based procedures enabling subretinal blood evacuation:

Pneumatic displacement (PD) performed with or without intravitreal injection of rtPA [2,4,5,6].

PPV combined with rtPA injection, administered either subretinally or intravitreally [2,3,4,5,6,7,8].

PPV with rtPA and anti-vascular endothelial growth factor (anti-VEGF) therapy, delivered subretinally or intravitreally [7].

PPV with subretinal injection of a “cocktail” comprising rtPA, anti-VEGF agent, and filtered air [6,7,8].

More radical procedures, such as retinectomy or autologous RPE-choroid grafts, which are less frequently described and typically reserved for particularly extensive or refractory cases [8].

Some reports have also described the use of tissue adhesives or other adjunctive substances aimed at limiting further hemorrhage expansion. The clinical characteristics and treatment outcomes of patients with SMH are summarized in Table 1.

#### 3.1.1. Pneumatic Displacement

PD is considered one of the less invasive therapeutic options for the management of SMH. The procedure involves intravitreal injection of an expansile gas, typically sulfur hexafluoride (SF6) or perfluoropropane (C3F8), which facilitates mechanical displacement of the hemorrhage from the fovea toward the peripheral retina. The gas may be administered alone or in combination with an intravitreal injection of rtPA. The recommended rtPA dosage ranges from 25 µg to 50 µg, diluted in 0.05 mL of balanced salt solution (BSS) or 0.9% saline. The rtPA used in this procedure is Actilyse (100 µg/mL) [4].

Pneumatic displacement (PD) performed with or without intravitreal injection of rtPA [2,4,5,6]. During PD, an anterior chamber paracentesis of approximately 0.3–0.5 mL is performed, followed by intravitreal injection of 0.3 mL of pure SF_6_ gas [4,5]. Some authors, however, report using SF_6_ at a concentration of 20% instead of pure gas [2].

This technique is primarily indicated for small, thin SMH, often in younger patients with post-traumatic hemorrhages and no underlying retinal pathology. Face-down positioning is recommended for approximately 5–7 days post-procedure to ensure optimal clot displacement [12].

#### 3.1.2. Pars Plana Vitrectomy

In PPV, precise removal of the vitreous body—including the central and peripheral vitreous as well as any epiretinal membranes—is critical. This is followed by the mechanical and/or pharmacological displacement or evacuation of subretinal blood. Patients most selected for PPV include those with large or thick hemorrhages, the presence of tractional membranes, concomitant RD, or active subretinal neovascularization. There is no absolute consensus regarding the optimal gauge for vitrectomy ports. However, most reports describe the use of 23-gauge (23G) instrumentation [1,3,5,6,9,10,11,13], with 25-gauge (25G) systems being used less frequently [5,8,14].

#### 3.1.3. Recombinant Tissue Plasminogen Activator

RtPA may be administered as a monotherapy or as part of a “pharmacological cocktail”. Combinations of rtPA with anti-VEGF agents have been described, as well as triple combinations including rtPA, anti-VEGF, and filtered air [6,7,8]. The use of subretinal air was first proposed and described by Mahmoud and Martel.

The primary rationale for using rtPA lies in its thrombolytic activity. Its use aids in the displacement and drainage SMH and may reduce the risk of iatrogenic injury to the RPE during surgery. RtPA can be administered either intravitreally or subretinal [1,2,3,4,5,6,7,8,9,10,11,14,15]. The diameter of the needle used for subretinal drug delivery is also critical. Given the trend toward micro incisional vitreoretinal surgery, surgeons increasingly utilize 41G microneedles [3,4,6,8,10,14], with common needle sizes ranging from 38G to 41G. There is also a report of successful subretinal injection using a 23G needle [1]. Subretinal injection may be performed manually by an assistant or via an automated injection system (e.g., DORC EVA, Alcon, Bausch and Lomb), which allows for precise control of injection speed using a foot pedal [3].

In pneumatic displacement (PD), intravitreal injection is the standard approach, whereas in pars plana vitrectomy (PPV), rtPA is more commonly delivered into the subretinal space. The retina—particularly the internal limiting membrane (ILM)—acts as a diffusion barrier for large molecules such as rtPA. Intravitreal rtPA demonstrates poor penetration through the retina into the subretinal space, resulting in limited efficacy. If the hemorrhage is very thin and in close apposition to the retina, partial clot dissolution may occur, but therapeutic outcomes appear to be inferior compared to subretinal rtPA administration. Combining intravitreal rtPA with an expansile gas (SF6 or C3F8) enhances the mechanical displacement of SMH [2,4,5,6,15]. This combined approach may be considered in cases of small SMH or in patients who are not suitable candidates for more invasive surgical interventions due to systemic contraindications.

Direct subretinal delivery of rtPA is widely regarded as the most effective approach, as it places the enzyme exactly at the site of hemorrhage [1,2,3,4,6,7,8,9,10,11,14]. To preserve foveal integrity, injections should be sited outside the central macula—ideally along the inferotemporal vascular arcade [3,6] or in areas of maximal central subfield thickness, where the greatest separation between retina and RPE minimizes the risk of iatrogenic trauma [14]. Chauhan et al. [1] demonstrated this technique by administering two subretinal injections—in the superotemporal and inferotemporal quadrants. Moreover, intraoperative OCT guidance has been shown to significantly enhance both the accuracy and safety of injection-site selection [6]. Reported subretinal rtPA concentrations range from 100 µg/mL [1,4,9] and 125 µg/mL [6,10] to 250 µg/mL [3,5,7], with some authors using up to 500 µg/mL [11], corresponding to total doses of 25–50 µg.

Animal models suggest that a subretinal rtPA dose up to 50 µg is safe, with higher amounts posing a risk of retinal toxicity [16]. Interestingly, Chauhan et al. [1] reported successful use of 60 µg of rtPA in a case of traumatic submacular hemorrhage, achieving improved best-corrected visual acuity despite exceeding the conventional limit. Subretinal rtPA may be injected alone or combined in a “cocktail” with anti-VEGF agents and filtered air [6,7,8]. The air bubble serves multiple functions: it mechanically displaces the hemorrhage inferiorly, counteracts red-blood-cell buoyancy, shields the fovea, sustains a localized retinal detachment to prolong drug contact, and even provides a transient oxygen reservoir for photoreceptors.

#### 3.1.4. Anti-VEGF

Several authors have demonstrated improved treatment outcomes with the addition of anti-VEGF therapy [2,3,4,6,7,10,11]. These agents may be administered intraoperatively or postoperatively on a *pro re nata* (PRN) basis. Subretinal administration of anti-VEGF agents—such as 2 mg/0.05 mL of aflibercept or 0.5 mg/0.05 mL of ranibizumab—allows direct targeting of the area where VEGF is produced. The use of fine-caliber needles (38–41G) has reduced the incidence of iatrogenic retinal detachments and proliferative vitreoretinopathy (PVR) [7].

Matias Iglicki et al. [7] compared the efficacy of intravitreal versus subretinal administration of aflibercept. Patients receiving subretinal aflibercept showed significantly greater improvements in BCVA and CST. This was attributed to enhanced VEGF suppression at the hemorrhage site following direct subretinal delivery. In contrast, intravitreal anti-VEGF reaches the subretinal space in lower concentrations. To enhance the subretinal penetration of anti-VEGF agents, ILM peeling may be performed [6,11,17]. It is also noteworthy that the half-life of anti-VEGF agents is shortened in vitrectomized eyes [6].

Management with intravitreal anti-VEGF monotherapy, even without additional surgical or pharmacologic interventions, may be an effective approach for promoting hemorrhage resolution and functional recovery in cases of extensive SMH secondary to AMD [18,19,20].

#### 3.1.5. Retinotomy, Retinectomy

In chronic, extensive SMH complicated by proliferative vitreoretinopathy or retinal detachment, PPV can be extended to include a retinotomy or retinectomy [8,13]. Retinectomy typically encompasses at least 180° of the temporal periphery near the ora serrata, allowing thorough removal of both the hemorrhage and the diseased RPE. The resultant RPE defect is often reconstructed with an autologous RPE–choroid graft harvested from healthy peripheral retina—most commonly the superotemporal quadrant [8,13]. Subretinal clot aspiration carries a high risk of RPE trauma from instrument manipulation and is therefore reserved for select cases. Following retinectomy, silicone oil remains the preferred endotamponade to ensure long-term retinal apposition [8,13].

#### 3.1.6. Endotamponade

Sulfur hexafluoride (SF_6_) and perfluoropropane (C_3_F_8_) are the most commonly employed endotamponade gases in PPV for SMH [2,4,6,8,9]. Choice of agent hinges on required tamponade duration, buoyant force and the patient’s risk profile. SF_6_ with its shorter intraocular longevity and lower expansion, is favored for smaller hemorrhages, in eyes at higher risk of steroid- or surgery-induced glaucoma, or when prolonged prone positioning is impractical. By contrast, C_3_F_8_ with its extended tamponade effect and greater upward force—is better suited to large or chronic SMHs that demand prolonged displacement from the foveal center. In less extensive SMH, SF_6_’s safety profile often makes it the preferred option. Filtered air has also been shown to provide effective, rapid tamponade with minimal toxicity [2,3,5,10,11]. In select cases—particularly those requiring retinectomy or managing complex subretinal pathology—silicone oil remains a useful, longer-lasting alternative [1,8,13].

### 3.2. Complications

The most common complication of SMH surgical treatments is recurrence of submacular hemorrhage. Pierre-Henry Gabrielle et al. [4] reported recurrent SMH in 2 eyes (5%) in the PPV group and in 6 eyes (15.79%) in the PD group. Vitreous opacities are also common complications accompanying extensive SMH. Evidence suggests that these are not directly caused by the treatment itself, but rather by extensive retinal damage due to massive hemorrhage. The weakened barrier function of the ILM of the retina may facilitate the migration of erythrocyte fragments into the vitreous cavity [21].

The most common complication after surgical treatment of SMH is recurrent submacular hemorrhage. Gabrielle et al. [4] reported recurrence rates of 5% after PPV and 15.8% after PD in their randomized trial. Across observational series, reported overall postoperative complication rates vary widely (approximately 5–25%) depending on technique and baseline disease severity [5,6,7,8]. The most frequently described complications are:

Recurrent submacular hemorrhage (5–20%)—reported in several series [4,5,6,7,8].

Vitreous hemorrhage (≈7–23%)—documented in Barzelay, Szeto and Boral and earlier PD series [5,6,8,22,23].

Retinal detachment (≈3–10%)—reported in Gabrielle, Boral and other surgical cohorts [3,4,8].

Transient intraocular pressure elevation (≈5–17%)—reported after both PPV and PD [4,6,8].

Cataract progression (≈10% after PPV; ≈5% after PD)—commonly reported after vitrectomy series, with [5,8,9] also documenting lens changes in real-world cohorts.

Cystoid macular edema (CME)—reported in real-world cohorts, e.g., Boral et al. [8].

Endophthalmitis (<1%)—rare and noted as potential risk in RCT and large series [4,6].

Pupillary block/angle closure and abnormal blood displacement have been described after PD in older series [22,24].

Rare toxic or exudative complications (e.g., exudative RD after high-dose tPA)—single case reports/series document this risk [25].

References supporting frequencies are given. Table 2 collates the key publications reporting complications associated with submacular hemorrhage (SMH) treatment.

## 4. Discussion

Over the years, the approach to treating SMH has shifted from active surgical removal of the hemorrhage—which carries a higher risk of iatrogenic damage—towards less invasive methods.

In cases of small, short-duration SMH, intravitreal administration of anti-VEGF agents, combined with pneumatic displacement when necessary, may be effective.

For medium and larger SMHs lasting up to approximately four weeks, a more invasive approach is recommended, such as PD ± rtPA administration, PPV with subretinal injection of rtPA ± anti-VEGF ± filtered air.

For large SMHs persisting beyond four weeks, complex surgical procedures involving mechanical removal of the SMH along with neovascular tissue beneath the retina are employed.

In clinical cases warranting such treatment, timely decision-making is crucial. The choice of treatment method depends largely on the underlying cause of SMH. Delaying surgical intervention worsens the prognosis for final visual acuity due to the toxic effects of ferritin released from erythrocytes, which damages photoreceptors. Additionally, the physical barrier formed by the presence of SMH separates the RPE and choroid from the photoreceptors, reducing nutrient diffusion [27].

However, the use of rtPA itself may contribute to toxic damage to the RPE. Clinical studies administering 50 μg tPA in SMH cases showed diffuse RPE changes and reduced electroretinogram amplitudes. It appears that therapy is safer at a concentration of 25 μg tPA [26]. In traumatic SMH, a single PD procedure performed within 30 days of hemorrhage onset yields optimal displacement and visual benefits [24].

Comparative studies yield nuanced insights into PD versus PPV approaches. Barzelay et al. [5] reported superior BCVA gains following PPV with subretinal rtPA versus PD with intravitreal rtPA in small-to-medium SMHs. In contrast, Bell et al. [15] found no significant BCVA difference between these approaches, though their cohort was neither stratified by SMH size nor uniformly treated with rtPA in the PD arm. Szeto et al. [6] further refined these comparisons by stratifying SMHs into small, medium, and massive categories and demonstrating that PPV with a subretinal three-drug cocktail (rtPA, anti-VEGF, and air) achieved complete SMH displacement 11.1-fold more often—and displacement to or beyond the vascular arcades 5.15-fold more often—than PD, along with greater central subfield thickness (CST) reduction. Both strategies, however, yielded clinically meaningful BCVA improvements.

Importantly, the absence of large, prospective randomized controlled trials directly comparing anti-VEGF monotherapy with combined surgical approaches remains a major limitation in the current literature. The ongoing pan-European phase III TIGER trial aims to address this gap by randomizing patients with recent, fovea-involving SMH to either standard anti-VEGF treatment or vitrectomy with subretinal tPA, gas tamponade, and adjunctive anti-VEGF therapy. Its results are expected to provide higher-level evidence to guide optimal treatment selection in SMH management. Long-term visual prognosis hinges on effective suppression of underlying neovascular AMD. A regimen of postoperative anti-VEGF on a pro re nata basis is widely endorsed [2,3,4,6,7,9,11]. Chang et al. [28] demonstrated that large-SMH patients undergoing PPV without subsequent anti-VEGF experienced initial BCVA gains that waned over time, whereas those receiving PRN injections maintained their visual improvements across follow-up. Similarly, Patikulsila et al. [9] and Said et al. [3] confirmed that adjunctive postoperative anti-VEGF therapy and control of AMD activity constitute the strongest predictors of durable, favorable visual outcomes.

Photodynamic therapy, particularly in combination with anti-VEGF, represents a well-established modality for managing polypoidal choroidal vasculopathy and achieving polyp regression. However, its use in the context of recent submacular hemorrhage remains controversial. Subretinal or submacular blood may preclude adequate visualization and precise laser delivery, and there are reports of significant rates of post-PDT bleeding. Therefore, while PDT may be considered as a complementary, adjunctive therapy in the peri- or postoperative phase in selected PCV cases, its application should be cautious and individualized, and further research is needed to evaluate its efficacy and safety in the specific setting of SMH [29].

## 5. Conclusions

The surgical technique for treating SMH must be individualized for each patient’s clinical condition. There are no definitive treatment guidelines for SMH. The factors most commonly influencing the choice of treatment method include the size of the SMH, its thickness, and the time elapsed from symptom onset to surgery. Currently, there is a trend toward minimally invasive approaches; however, in cases of large, longstanding SMHs, invasive methods are beneficial. Our study summarizes detailed surgical techniques along with descriptions of procedures used over the past five years.

## Figures and Tables

**Table 1:** This table summarizes key characteristics and outcomes from various clinical studies investigating treatments for submacular hemorrhage (SMH), mainly due to neovascular age-related macular degeneration (nAMD), polypoidal choroidal vasculopathy (PCV), and other retinal conditions.

Study	Reason	SMH Size	Time of Symptoms	Number of Treated Eyes	Treatment	Change in VA (LogMAR, Unified)	Follow-Up Period
Pierre-Henry Gabrielle et al. [4]	nAMD	>2 DD, CST > 100 μm	≤14 days	90	PPV + subretinal tPA + intravitreal anti-VEGF PD + intravitreal tPA + intravitreal anti-VEGF	PPV group +16.8 letters ≈ +0.34 LogMAR PD group +16.4 letters ≈ +0.33 LogMAR At month 6, BCVA was similar in both groups.	6 months
Direk Patikulsila et al. [9]	nAMD, PCV	>4 DD (mean 6.2)	Mean 15.1 days	9	PPV + subretinal tPA ± antiVEGF intravitreal	1.9 → 1.1 (Δ = 0.8 LogMAR improvement)	16.1 months
Subhendu Kumar Boral et al. [8]	nAMD, PCV	>4 DD	- Group A >4 weeks - Group B 4–8 weeks - Group C >8 weeks	103	Group A PPV + subretinal rtPA, antiVEGF, filtered air Group B PPV + subretinal rtPA and retinotomy/retinectomy Group C PPV + subretinal BBS, retinectomy + autotransplantation of RPE-choroid flap	Group A: 2.09 → 0.96; B: 2.46 → 1.50; C: 2.69 → 1.03	Mean 11.12 months
Yasmin Ali Said et al. [3]	nAMD, macroaneurysm	>1 DD	≤14 days	93	PPV + subretinal tPA	0.06 → 0.10	6 weeks
Agnieszka Nowosielska et al. [10]	nAMD	≤1.5 DD	Mean 19.6 days	10	PPV + subretinal tPA + intravitreal antiVEGF	Before surgery VA “0.05/hand movement” → 0.35	6 months
Smt. Kanuri Santhamma et al. [1]	traumatic	-	-	1	PPV + retinotomy + 60 µg tPA	Before surgery finger counting After 1 month 0.60 After silicone oil removal 0.48	-
Yunxi Ma et al. [11]	PCV, nAMD	2–5 DD	Mean 18.07 days	14	PPV + subretinal rtPA + intravitreal antiVEGF	Before surgery 1.68 7 days after surgery 1.55 1 month after 1.38 3 months after 1.23 6 months after 1.18	6 months
Anna Hillenmayer et al. [2]	nAMD, Valsalva retinopathy, macroaneurysm, peripheral exudative hemorrhagic retinopathy	10–74 DD	≤14 days	201	Group 1 PD + intravitreal rtPA injection Group 2 PD + intravitreal rtPA injection ± PPV + subretinal rtPA Group 3 PPV + subretinal rtPA Group 4 PPV Group 5 PPV ± subretinal rtPA + subretinal lavage	1.7 → 1.4	Mean 4 months
Matias Iglicki et al. [7]	nAMD	Not reaching the vascular arches	Mean 1.26 days	80	PPV + tPA + gas subretinal + VEGF submacular or intravitreal	Intravitreal group 0.749 → 0.687 Submacular 0.651 → 0.473	24 months
Aya Barzelay et al. [5]	nAMD	Small ≤ 20 mm^2^ Medium 20–50 mm^2^ Large ≥ 50 mm^2^	PPV group 4.92 days PD group 3.79 days	150	PPV with tPA subretinal PD with tPA intravitreal	Small PPV gains ≈ +0.77 vs. PD 0.04 Medium: no difference	12 months
Simon K. H. Szeto et al. [6]	PCV nAMD	Arcade-based classification	PPV 10.65 days PD 5.53 days	63	PD + tPA intravitreal ± antiVEGF intravitreal PPV with tPA, anti-VEGF and subretinal air	PD 1.46 → 0.67 PPV 1.62 → 0.91	12 months

Abbreviations: SMH, submacular hemorrhage; DD, disk diameter; CST, central subfield thickness; nAMD, neovascular age-related macular degeneration; PCV, polypoidal choroidal vasculopathy; PPV, pars plana vitrectomy; PD, pneumatic displacement; rtPA, recombinant tissue plasminogen activator; anti-VEGF, anti-vascular endothelial growth factor therapy; VA, visual acuity; BCVA, best-corrected visual acuity; logMAR, logarithm of the minimum angle of resolution.

**Table 2:** The table summarizes multiple studies investigating surgical, pars plana vitrectomy (PPV), pneumatic displacement (PD) treatments for submacular hemorrhage (SMH), focusing on underlying conditions, number of treated eyes and observed complications. The abbreviation NR indicates that specific information was not reported by the authors.

Study	Fujikawa M et al. [26]	Pierre-Henry Gabrielle et al. [4]	Direk Patikulsila et al. [9]	Subhendu Kumar Boral et al. [8]	Yasmin Ali Said et al. [3]	Anna Hillenmayer et al. [2]	Matias Iglicki et al. [7]	Aya Barzelay et al. [5]	Simon K. H. Szeto et al. [6]
Reason	NR	nAMD	nAMD, PCV	nAMD, PCV	nAMD, macroaneurysm	nAMD, Valsalva retinopathy, macroaneurysm, peripheral exudative hemorrhagic retinopathy	nAMD	nAMD	nAMD PCV
Number of treated eyes	NR	PPV 45 PD 44	9	103	93	201	80	150	63
Recurrent SMH	3.3%	PPV 2 PD 6	1	A 4.84% B 12.9% C 10%	11%	NR	NR	PPV 13.6% PD 19.4%	PPV 18.2% PD 5.3%
Rhegmatogenous retinal detachment	3.3%	PPV 4 PD 0	0	A 3.23% B 0% C 10%	6.6%	1%	NR	NR	PPV 8.7% PD 5%
Vitreous hemorrhage	23.3%	PPV 5 PD 5	1	A 6.45% B 0% C 0%	7.7%	6%	NR	PPV 4.5% PD 16.7%	PPV 30.4% PD 17.5%
Retinal rupture	NR	NR	0	NR	2.2%	NR	NR	NR	PPV 8.7% PD 7.5%
Endophthalmitis	NR	PPV 0 PD 0	NR	NR	NR	NR	NR	NR	NR
Elevated IOP	NR	PPV 2 PD 2	0	A 0% B 0%	NR	7%	0	NR	PPV 17.4% PD 7.5%
Hypotony	NR	NR	NR	B 3.23% C 20%	NR	NR	NR	NR	NR
Cataract	NR	PPV 2 PD 2	3/7	Present number NR	NR	NR	NR	PPV 9.8% PD 11.4%	NR
Macular hole	NR	PPV 1 PD 0	0	A 6.45%	NR	NR	0	NR	PPV 4.3% PD 0%
Epiretinal membrane	NR	NR	1	A 0% B 16.13% C 0%	NR	NR	NR	NR	NR

Abbreviations: nAMD, neovascular age-related macular degeneration; PCV, polypoidal choroidal vasculopathy; SMH, submacular hemorrhage; PPV, pars plana vitrectomy; PD, pneumatic displacement; IOP, intraocular pressure.

## Data Availability

All data generated or analyzed during this study are included in this published article.

## Abbreviations

SMH	submacular hemorrhage
nAMD	neovascular age-related macular degeneration
rtPA	recombinant tissue plasminogen activator
PCV	polypoidal choroidal vasculopathy
PPV	pars plana vitrectomy
PD	pneumatic displacement
IOP	intraocular pressure
DD	disk diameter
OCT	optical coherence tomography
Anti-VEGF	anti-vascular endothelial growth factor
RPE	retinal pigment epithelium
SF6	sulfur hexafluoride
C3F8	perfluoropropane
25G	25-gauge
23G	23-gauge
ILM	internal limiting membrane
